# The effect of metformin therapy on incidence and prognosis in prostate cancer: A systematic review and meta-analysis

**DOI:** 10.1038/s41598-018-38285-w

**Published:** 2019-02-18

**Authors:** Kancheng He, Huating Hu, Senlin Ye, Haohui Wang, Rongrong Cui, Lu Yi

**Affiliations:** 1 Department of Urology, The Second Xiangya Hospital, Central South University, Changsha, 410011 China; 2 The First Hospital of Hunan University of Chinese Medicine, Changsha, 410007 China; 3 Institute of Metabolism and Endocrinology, The Second Xiangya Hospital, Central South University, Changsha, 410011 China

## Abstract

The relationship between metformin and prostate cancer (PCa) remains controversial. To clarify this association, the PubMed, Embase and Cochrane library databases were systematically searched from their inception dates to May 23, 2018, using the keywords “metformin” and “prostate cancer” to identify the related studies. The results included incidence, overall survival (OS), PCa-specific survival (CSS) and recurrence-free survival (RFS), which were measured as hazard ratios (HR) with a 95% confidence interval (95% CI) using Review Manager 5.3 software. A total of 30 cohort studies, including 1,660,795 patients were included in this study. Our study revealed that metformin treatment improves OS, CSS and RFS in PCa (HR = 0.72, 95% CI: 0.59–0.88, P = 0.001; HR = 0.78, 95% CI: 0.64–0.94, P = 0.009; and HR = 0.60, 95% CI: 0.42–0.87 P = 0.006, respectively) compared with non-metformin treatment. However, metformin usage did not reduce the incidence of PCa (HR = 0.86, 95% CI: 0.55–1.34, P = 0.51). In conclusion, compared with non-metformin treatment, metformin therapy can significantly improve OS, CSS and RFS in PCa patients. No association was noted between metformin therapy and PCa incidence. This study indicates a useful direction for the clinical treatment of PCa.

## Introduction

Prostate cancer (PCa) is the second leading cause of malignancy deaths among men in the United States. Approximately 164,690 American males were diagnosed with PCa in 2017, and 29,430 will die of this disease^1^. Given the wide used of earlier detection modalities and advances in treatment, the incidence and mortality of PCa exhibit a sharp reductions^1,2^. However, Boorjian *et al*.^3^ reported that up to 40% of PCa patients faced challenges of cancer recurrence or progression during long-term follow-up.

Metformin, an oral biguanide mainly used to treat type 2 diabetes, has demonstrated anti-neoplastic effects in several types of solid tumours and hormone-sensitive tumours, such as colon cancer, pancreatic cancer and breast cancer^4–6^. Metformin inhibits cancer proliferation by activating the AMPK pathway and suppressing the expression of genes involved in mitosis^7,8^. Given that hyperinsulinaemia is associated with an increased risk of colorectal and breast cancer, a poor prognosis is often noted^9^. As an insulin sensitizer, metformin exhibits indirect antitumour effects by reducing insulin levels through the inhibition of hepatic gluconeogenesis. However, the effects of metformin use in prostate cancer, an analogous hormonally sensitive cancer in men, remain controversial. Several studies^10,11^ demonstrated that metformin reduces the risk of prostate cancer incidence and improve PCa outcomes. In contrast, other studied reported negative outcomes.

Given the association between metformin and cancer incidence, the prognosis of prostate cacncer remain unclear. In this study, we evaluated the incidence and prognostic value of metformin in prostate cancer.

## Result

**Figure 1:**
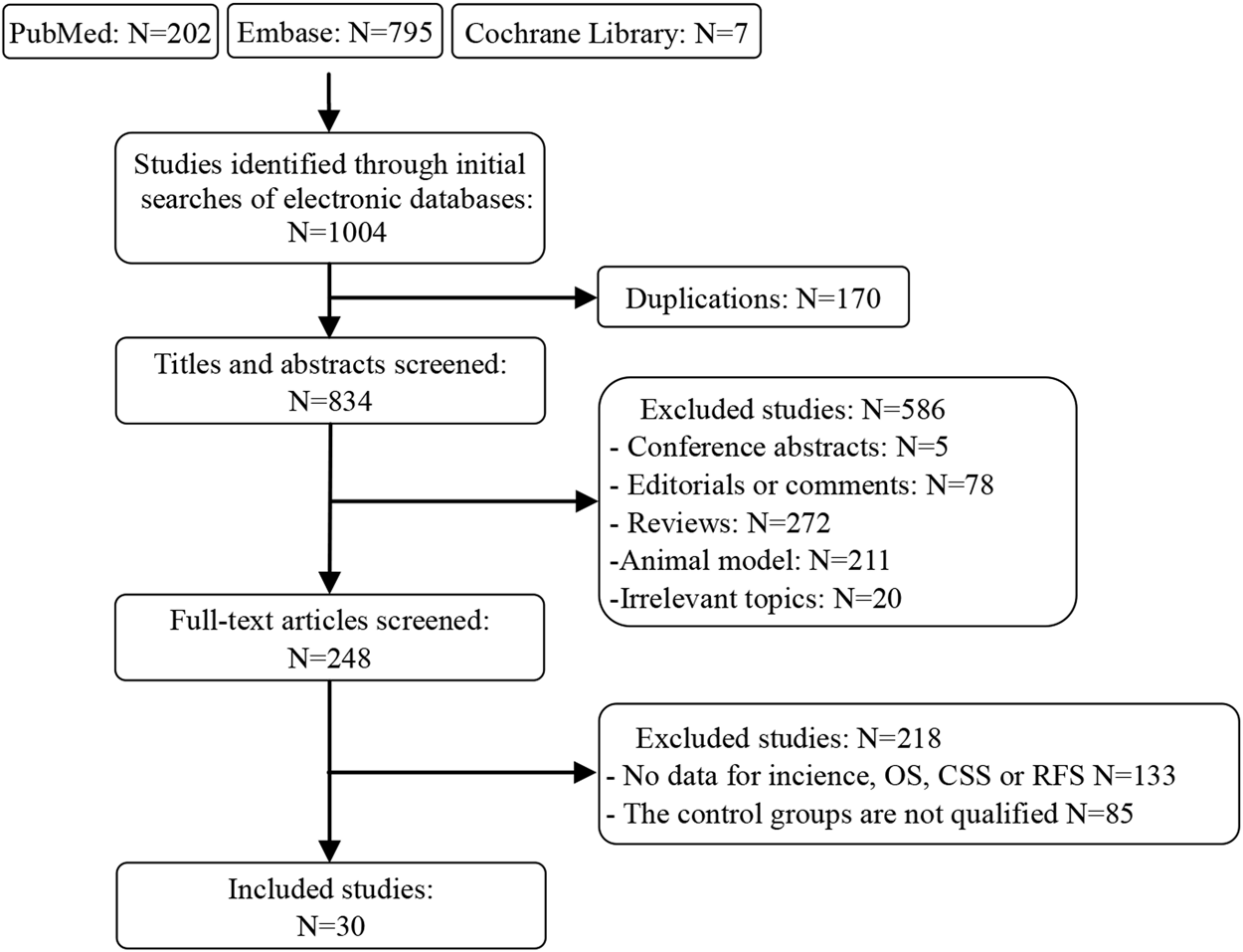
Literature search and screening process.

In total, 1004 publications were identified for eligibility through a literature search. After removing the duplicate studies and reviewing titles and abstracts, 30 studies and 1,660,795 individuals were included in our meta-analysis (Fig. 1).

### Study characteristics

The baseline characteristics of all included studies are presented Table 1. Studies were published between 2012 and 2017. There were 12, 14, 7 and 8 publications associated with incidence, OS, CSS, and RFS, respectively. 19 studies were performed in the United States, 8 in Europe, 2 in Asia and 1 in Australia. Four studies were conducted in patients with prostatectomy, 4 with radiotherapy, 1 with ADT, and 1 with docetaxel. Eight studies included a mixture of these PCa treatments. Newcastle-Ottawa Scale (NOS) was used to assess the methodological quality of included studies, which ranged from 6 to 9 (Table 2).

**Table 1:** Basic characteristic of 30 studies included in Meta-analysis.

First author(year)	Study region	Inclusion time	Treatment	Metformin user/total patients	Study design	Study setting	Outcomes	Article type
Mayer 2017^16^	Canada	2005–2012	Docetaxel	359/2832	Retrospective Cohort	Population-base	OS CSS	Full
Zaorsky 2017^57^	USA	1998–2013	Radiotherapy	251/3217	Retrospective Cohort	Hospital-base	OS CSS RFS	Full
Richards 2017^17^	USA	2000–2015	ADT	14517/87344	Retrospective Cohort	Population-base	OS CSS	Abstract
Jarrard 2017^58^	USA	N/A	Mix therapy	68/788	Prospective Cohort	Population-base	OS	Abstract
Haggstrom 2017^39^	Sweden	2006–2013	N/A	10224/612846	Prospective Cohort	Population-base	Incidence	Full
Chen 2017^49^	Canada	1994–2012	N/A	35829/44172	Retrospective Cohort	Population-base	Incidence	Full
Haring 2017^43^	Finland	1995–2009	N/A	8989/78615	Prospective Cohort	Population-base	Incidence	Full
Chong 2016^59^	USA	N/A	Mix therapy	138/287	Retrospective Cohort	Hospital-base	OS RFS	Full
Joentausta 2016^60^	Finland	1995–2009	Prostatectomy	133/1314	Retrospective Cohort	Population-base	OS RFS	Full
Wang 2016^48^	USA	2003–2012	N/A	29805/76733	Retrospective Cohort	Population-base	Incidence	Full
Raval 2016^40^	USA	2008–2009	N/A	938/2652	Retrospective Cohort	Population-base	Incidence	Full
Xu 2015^61^	USA	1995–2010	Mix therapy	vanderbilt: 218/32415 Mayo: 3029/79258	Retrospective Cohort	Hospital-base	OS	Full
Randazzo 2015^62^	Swizerland	1998–2003	Mix therapy	150/4314	Prospective Cohort	Population-base	OS	Full
Lee 2015^63^	Korea	2006–2013	Prostatectomy	135/746	Retrospective Cohort	Hospital-base	RFS	Full
Reznicek 2015^64^	USA	2002–2010	Mix therapy	N/A/1155	Retrospective Cohort	Hospital-base	OS	Abstract
Lu-Yao 2015^52^	USA	2007–2009	Mix therapy	N/A	Retrospective Cohort	Population-base	CSS	Abstract
Danzig 2015^65^	USA	1987–2010	Prostatectomy	98/767	Retrospective Cohort	Hospital-base	RFS	Full
Nordstrom 2015^41^	Sweden	2007–2012	N/A	7678/185667	Retrospective Cohort	Population-base	Incidence	Full
Feng 2015^42^	USA	N/A	N/A	194/693	Prospective Cohort	Population-base	Incidence	Full
Rieken 2014^66^	USA and Europa	2000–2011	Prostatectomy	287/6486	Retrospective Cohort	Hospital-base	RFS	Full
Bensimon 2014^54^	UK	1998–2009	Mix therapy	242/935	Retrospective Cohort	Population-base	OS CSS	Full
Spratt 2014^55^	Canada	1992–2008	Radiotherapy	157/319	Retrospective Cohort	Hospital-base	OS CSS RFS	Full
Taira 2014^21^	USA	1995–2010	Radiotherapy	126/2298	Retrospective Cohort	Hospital-base	OS RFS	Full
But 2014^53^	Finland	1997–2010	N/A	1188/23394	Retrospective Cohort	Population-base	Incidence	Full
Habel 2014^51^	USA	1997–2009	N/A	N/A	Retrospective Cohort	Population-base	Incidence	Abstract
Onitilo 2014^67^	Australia	1995–2009	N/A	5679/9468	Retrospective Cohort	Population-base	Incidence	Full
Tseng 2014^68^	China	1998–2002	N/A	186212/395481	Retrospective Cohort	Population-base	Incidence	Full
Zannella 2013^69^	Canada	1996–2012	Radiotherapy	114/504	Retrospective Cohort	Hospital-base	RFS	Full
Margel 2013^70^	USA	1997–2008	Mix therapy	1251/3837	Retrospective Cohort	Population-base	OS CSS	Full
Magliano 2012^71^	Australian	1993–2010	N/A	N/A/2258	Retrospective Cohort	Population-base	Incidence	Full

**Table 2:** Methodological quality of the 30 studies base on the Newcastle-Ottawa Scale for studies.

First author(year)	Study design	Selection	Comparability	Assessment of outcome	Total quality scores
Mayer 2017	Cohort	***	**	**	7
Zaorsky 2017	Cohort	**	**	***	7
Richards 2017	Cohort	**	**	**	6
Jarrard 2017	Cohort	***	**	**	7
Haggstrom2017	Cohort	****	**	***	9
Chen 2017	Cohort	***	**	**	7
Haring 2017	Cohort	****	**	***	9
Chong 2016	Cohort	****	**	*	7
Joentausta 2016	Cohort	***	**	**	7
wang 2016	Cohort	***	**	**	7
Raval 2016	Cohort	***	**	***	8
Xu 2015	Cohort	****	**	***	9
Randazzo 2015	Cohort	***	**	***	8
Lee 2015	Cohort	****	**	***	9
Reznicek 2015	Cohort	****	**	**	8
Lu-Yao 2015	Cohort	****	**	*	7
Danzig 2015	Cohort	***	**	*	6
Nordstrom 2015	Cohort	**	**	***	7
Feng 2015	Cohort	***	**	**	7
Rieken 2014	Cohort	****	**	***	9
Bensimon 2014	Cohort	***	**	**	7
Spratt 2014	Cohort	****	**	**	8
Taira 2014	Cohort	****	**	***	9
But 2014	Cohort	***	**	**	7
Habel 2014	Cohort	***	**	****	9
Onitilo 2014	Cohort	***	**	***	8
Tseng 2014	Cohort	**	**	***	7
Zannella 2013	Cohort	***	**	**	7
Margel 2013	Cohort	***	**	***	8
Magliano 2012	Cohort	***	**	**	7

### Metformin therapy and PCa overall survival

**Figure 2:**
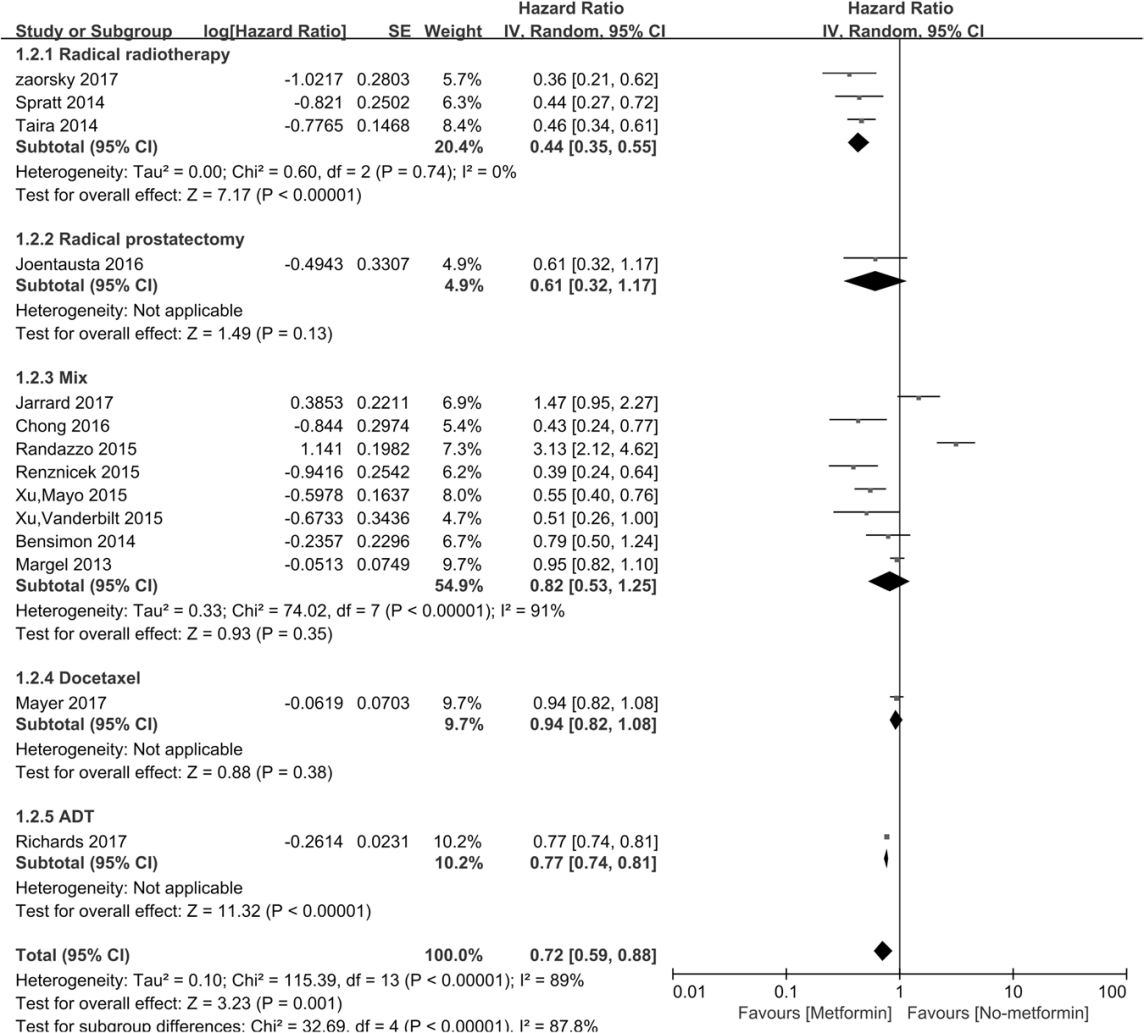
Forest plot for the pooled analyses of the association between metformin use and OS of the PCa patients, who accept prostatectomy, radiotherapy, mixed therapy, Docetaxel and ADT.

Figure 2 indicated that incidence of PCa was assessed in 14 studies. The HR for PCa patients taking metformin compared with those not taking metformin was 0.72 [95% *CI*: 0.59∼0.88], *P* = 0.001. Interstudy heterogeneity was noted (*I*^2^ = 89%, *P* < 0.00001). Metformin therapy improved the OS of PCa patients who accepted radiotherapy (n = 3, *HR* = 0.44, [95% *CI*: 0.35∼0.55], *P* < 0.00001). The subgroup studies consist of study region, study design, sample sizes, diabetic only, study setting and cumulative duration (Table 3).

**Table 3:** Subgroup analysis of PCa overall survival.

Items	Test for Heterogeneity		Include Study	Test for Overall effect		HR	95% *CI*
	*I* ^2^	*P*		*Z*	*P*		
**Study region**							
USA/Canada	85%	<0.00001	11	4.66	<0.00001	0.65	0.54 to 0.78
Europe	93%	<0.00001	3	0.29	0.77	1.17	0.40 to 3.37
**Study design**							
Prospective	85%	0.01	2	2.04	0.04	2.16	1.03 to 4.53
Retrospective	81%	<0.00001	12	5.54	<0.00001	0.62	0.53 to 0.74
**Sample size**							
<10000	90%	<0.00001	11	1.99	0.05	0.73	0.54 to 1.00
≥10000	64%	0.06	3	2.91	0.004	0.65	0.49 to 0.87
**Diabetic only**							
Yes	85%	<0.0001	5	2.48	0.01	0.55	0.34 to 0.88
No	91%	<0.00001	9	1.61	0.11	0.80	0.62 to 1.05
**Study setting**							
Hospital-base	0%	0.79	7	9.39	<0.00001	0.46	0.39 to 0.54
Population-base	91%	<0.00001	7	0.45	0.66	1.06	0.83 to 1.35
**Cumulative duration**							
≤1 yr	0%	0.57	2	7.39	<0.00001	0.88	0.85 to 0.91
1–3 yr	0%	0.42	2	5.38	<0.00001	0.93	0.91 to 0.95

### Metformin therapy and PCa-specific survival

**Figure 3:**
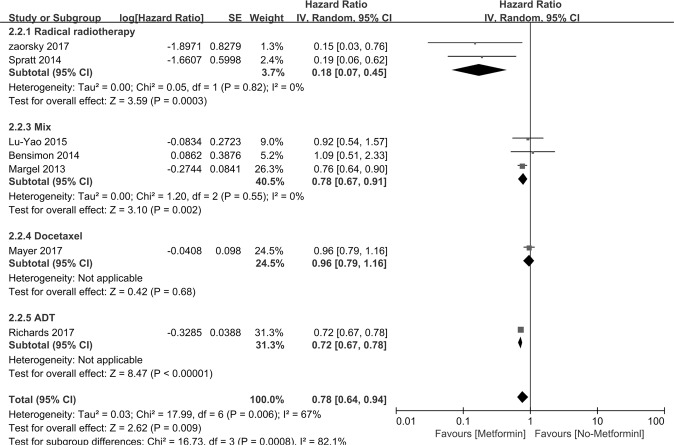
Forest plot for the pooled analyses of the association between metformin use and CSS of the PCa patients, who accept radiotherapy, mixed therapy, Docetaxel and ADT.

Figure 3 indicates that CSS was assessed in 7 studies. The HRs for CSS in PCa patients taking metformin compared with those not taking metformin was 0.78 [95% *CI:* 0.64∼0.94], *P* = 0.009. Interstudy heterogeneity was noted (*I*^2^ = 67%, *P* = 0.006). Metformin therapy improved the CSS of PCa patients who accepted radiotherapy or mix treatment (n = 2 *HR* = 0.18, [95% *CI*: 0.07∼0.45], *P* = 0.0003; n = 3 *HR* = 0.78, [95% *CI*: 0.67∼0.91], *P* = 0.002 respectively). The subgroup studies consist of study region, study design, sample sizes, diabetic only and study setting (Table 4).

**Table 4:** Subgroup analysis of PCa specific survival.

Items	Test for Heterogeneity		Include Study	Test for Overall effect		HR	95% *CI*
	*I* ^2^	*P*		*Z*	*p*		
**Study region**							
USA/Canada	71%	0.004	6	2.72	0.007	0.76	0.63 to 0.93
Europe	/	/	1	0.22	0.82	1.09	0.51 to 2.33
**Study design**							
Prospective	/	/	0	/	/	/	/
Retrospective	67%	0.006	7	2.62	0.009	0.78	0.64 to 0.94
**Sample size**							
<10000	72%	0.007	5	1.72	0.08	0.74	0.52 to 1.04
≥10000	0%	0.37	2	8.43	<0.00001	0.72	0.67 to 0.78
**Diabetic only**							
Yes	58%	0.1	3	1.11	0.27	0.70	0.37 to 1.32
No	77%	0.004	4	1.73	0.08	0.78	0.58 to 1.03
**Study setting**							
Hospital-base	0%	0.82	2	3.59	0.0003	0.18	0.07 to 0.45
Population-base	55%	0.06	5	3.01	0.003	0.81	0.70 to 0.93

### Metformin therapy and PCa recurrence free survival

**Figure 4:**
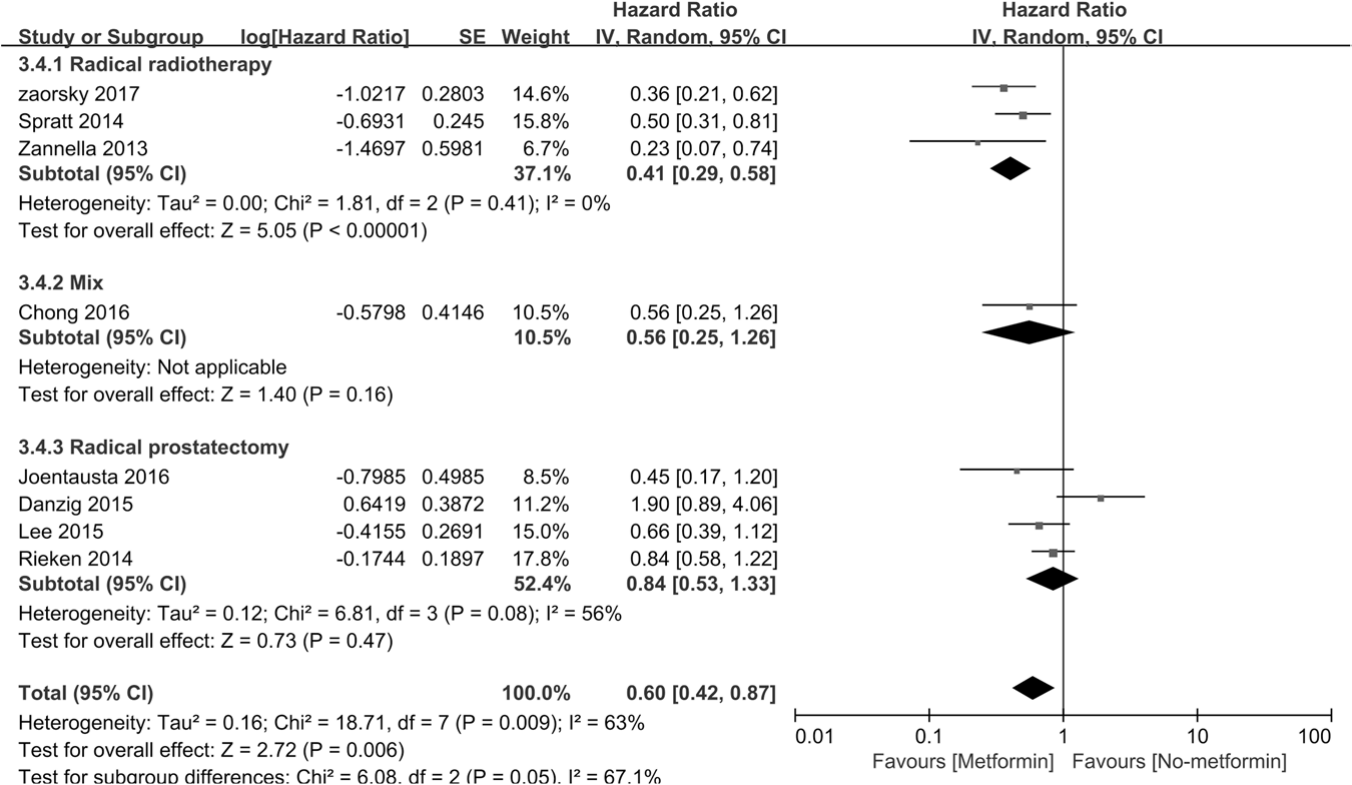
Forest plot for the pooled analyses of the association between metformin use and RFS of the PCa patients, who accept prostatectomy, radiotherapy, and mixed therapy.

Figure 4 indicates that RFS was assessed in 8 studies. The HRs for RFS in PCa patients taking metformin compared with those not taking metformin was 0.60, [95% *CI*: 0.42∼0.87] *P* = 0.006. Interstudy heterogeneity was noted (*I*^2^ = 63%, *P* = 0.009). In the subgroup of basic treatment, metformin therapy improved the RFS of PCa patients who accepted radiotherapy (n = 3 *HR* = 0.41, [95% *CI*: 0.29∼0.58], *P* < 0.00001). The subgroup studies consist of study region, sample sizes, diabetic only, study setting and study design (Table 5).

**Table 5:** Subgroup analysis of PCa recurrence-free survival.

Items	Test for Heterogeneity		Include Study	Test for Overall effect		HR	95% *CI*
	*I* ^2^	*P*		*Z*	*p*		
**Study region**							
USA/Canada	73%	0.005	5	1.92	0.05	0.55	0.30 to 1.01
Europe	27%	0.24	2	1.21	0.23	0.73	0.44 to 1.22
Asia	/	/	1	1.54	0.12	0.66	0.39 to 1.12
**Sample size**							
<10000	63%	0.009	8	2.72	0.006	0.60	0.42 to 0.87
≥10000	/	/	/	/	/	/	/
**Diabetic only**							
Yes	84%	0.002	3	0.67	0.51	0.71	0.26 to 1.93
No	39%	0.16	5	3.02	0.003	0.59	0.42 to 0.83
**Study setting**							
Hospital-base	67%	0.006	7	2.39	0.02	0.62	0.42 to 0.92
Population-base	/	/	1	1.6	0.11	0.45	0.17 to 1.20
**Study design**							
Prospective	/	/	0	/	/	/	/
Retrospective	63%	0.009	8	2.72	0.006	0.60	0.42 to 0.87

### Metformin therapy and incidence of PCa

**Figure 5:**
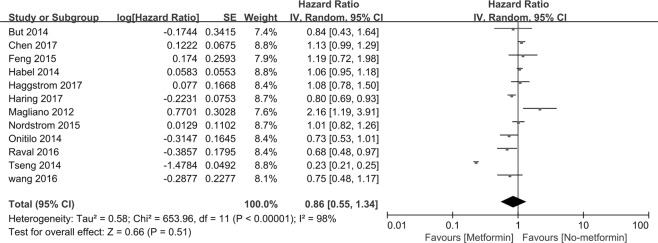
Forest plot for the pooled analyses of the association between metformin use and incidence of the PCa.

Figure 5 indicates that incidence of PCa was assessed in 12 studies. The HR for PCa patients taking metformin compared with those not taking metformin was 0.86 [95% *CI*: 0.55∼1.34], *P* = 0.51. Interstudy heterogeneity was noted (*I*^2^ = 98%, *P* < 0.00001). In our subgroup, 6 studies are classified according to their participants’ race, including African American, Hispanic/Latino, non-Hispanic white and Asian. Non-Hispanic whites with metformin therapy exhibit a reduced incidence of PCa (*HR* = 0.86, [95% *CI*: 0.76∼0.98], *P* = 0.02). No associations were found between metformin usage and African Americans, Hispanic/Latinos and Asians. The subgroup studies consist of study region, sample sizes, race, duration of metformin therapy, cumulative dose of metformin, Gleason of PCa, advanced PCa, diabetic only and cumulative duration. All subgroup analyses did not reveal any benefits for reducing the incidence of PCa (Table 6).

**Table 6:** Subgroup analysis of Incidence.

Items	Test for Heterogeneity		Include Study	Test for Overall effect		HR	95% *CI*
	*I* ^2^	*P*		*Z*	*p*		
**Study region**							
USA/Canada	64%	0.02	6	0.63	0.53	0.95	0.82 to 1.11
Europe	37%	0.19	4	1.08	0.28	0.91	0.78 to 1.08
Asia	/	/	1	30.05	<0.00001	0.23	0.21 to 0.25
Australia	/	/	1	2.54	0.01	2.16	1.19 to 3.91
**Study design**							
Prospective	54%	0.11	3	0.45	0.66	0.94	0.73 to 1.22
Retrospective	99%	<0.00001	9	0.64	0.53	0.84	0.48 to 1.45
**Sample size**							
<10000	78%	0.004	4	0.03	0.97	1.01	0.64 to 1.58
≥ 10000	99%	<0.00001	8	0.79	0.43	0.80	0.46 to 1.39
**Race**							
African American	26%	0.24	2	0.45	0.65	1.04	0.88 to 1.23
Latino/Hispanic	94%	<0.0001	2	0	1.00	1.00	0.40 to 2.52
Non-Hispanic while	49%	0.16	2	2.33	0.02	0.86	0.76 to 0.98
Asian	100%	<0.00001	2	0.85	0.4	0.51	0.11 to 2.43
**Gleason of PCa**							
Gleason ≥7	0%	0.38	2	1.76	0.08	1.25	0.98 to 1.59
Gleason <7	/	/	1	0.03	0.97	1.01	0.57 to 1.80
**Cumulative dose of Metformin**							
First tertile of metformin use	90%	<0.0001	3	0.89	0.37	0.88	0.67 to 1.16
Second tertile of metformin ues	98%	<0.00001	3	1.16	0.25	0.7	0.39 to 1.8
Third tertile of metformin use	99%	<0.00001	3	1.15	0.25	0.53	1.18 to 1.56
**Duration of metformin use**							
<2 yr	82%	0.0002	5	1.28	0.20	0.86	0.69 to 1.08
2–5 yr	96%	<0.00001	4	0.84	0.40	0.82	0.50 to 1.32
≥5 yr	98%	<0.00001	4	1.09	0.28	0.59	0.23 to 1.53
**Diabetic only**							
Yes	99%	<0.00001	9	0.60	0.55	0.84	0.48 to 1.48
No	56%	0.10	3	0.71	0.47	0.92	0.75 to 1.15

### Assessment of heterogeneity

There was evidence of considerable heterogeneity in OS (*I*^2^ = 89%, *P* < 0.00001), CSS (***I***^2^ = 67%, ***P*** = 0.006), RFS (***I***^2^ = 63%, ***P*** = 0.009) and incidence of PCa (*I*^2^ = 98%, *P* < 0.00001). Subgroup analyses investigating potential sources of heterogeneity demonstrated that study region, study design, study setting, sample size and diabetic only were not significantly associated with the heterogeneity in this meta-analysis. We also conducted a sensitivity analysis in which one study was removed at a time and found that the Zaosky 2017 study was the source of heterogeneity in the meta-analysis for RFS. When the study by Zaosky 2017 was removed, the heterogeneity in RFS decreased (*I*^2^ = 57%, *P* = 0.03), and the results remained stable (HR = 0.66, 95% CI: 0.45–0.96). In the group of incidence, Tseng’s 2014 study was the source of statistical heterogeneity in the meta-analysis. When the study by Tseng 2014 was removed, the heterogeneity in incidence decreased (*I*^2^ = 67%, *P* = 0.0007), and a meta-analysis of incidence results remained stable (HR = 0.96, 95% CI: 0.84–1.10). When the abovementioned studies were removed, the meta-analysis of RFS and incidence demonstrated statistical robustness. No study markedly affected the heterogeneity in the group of OS and CSS. This sensitivity analysis confirms the robustness of our results.

### Publication bias

Egger’s and Begg’s tests revealed the possibility of publication bias for OS (0.677), CSS (0.816), RFS (0.526) and incidence (0.284). No obvious publication bias was noted in our analysis.

## Discussion

In the past few years, the controversial results of metformin in the incidence and prognosis of PCa have been increasing. Increasing experimental research reports that metformin exhibits its own advantages in PCa treatment *in vitro*. Comstock *et al*.^12^ reported that the cyclin D1 pathway was related to PCa cell cycle progression and androgen-dependent transcription. Metformin inhibits PCa cell proliferation by reducing cyclin D1 activity^13^. Metformin also reduces PCa cell viability and enhances apoptosis by downregulating androgen receptors in both androgen-dependent and androgen-independent prostate cancers^14^. Metformin activates the AMP-activated protein kinase (AMPK), which inhibits mTOR signalling. Given that mTOR is overexpressed in PCa, metformin reduces PCa growth^15^. In clinical research, the effect of metformin on PCa is uncertain. Mayer *et al*.^16^ reported that metformin used with docetaxel did not affect castration-resistant PCa-specific survival and overall survival. However, another study^17^ reported that ADT with metformin prolongs advanced PCa-specific survival and overall survival. To clarify the relationship between metformin and PCa, a total of 30 cohort studies encompassing 1,660,795 individuals were included in our present systematic review and meta-analysis.

In our meta-analysis of PCa and metformin, we found that PCa patients who use metformin exhibited OS, CSS and RFS benefits compared with PCa patients who did not take metformin. This result is similar with previous meta-analysis, which reported that metformin was useful for OS and RFS^18,19^. However, Stopsack *et al*.^18^ included 4 studies and Xiao *et al*.^19^ included 6 studies. Thus, the meta-analysis was limited due to low event numbers in studies reporting CSS. However, unlike the previous meta-analysis, we found that metformin therapy was associated with CSS by including 7 studies. We first grouped the included studies based on basic treatment (prostatectomy, radiotherapy, ADT, etc.), metformin dose, and duration of metformin therapy and observed that patients who accepted both radical radiotherapy and metformin therapy had a significant improvement in OS, CSS and RFS in our meta-analysis. An *in vitro* study reported that metformin enhanced ionizing radiation activation of AMPK in PC-3 cells and reduced the surviving fraction of PC-3^20^. These results demonstrated that metformin induced radiosensitizing effects. Interestingly, many studies reported^21–23^ that the prognosis of PCa patients who accepted prostatectomy was not associated with metformin use, dose or duration of use. These contradictory data between prostatectomy and radiotherapy were significant. Pre-operative ADT exhibited no survival benefit in men accepting prostatectomy^24,25^, but was benefical in radical radiotherapy^26^. Taira *et al*.^21^ hypothesized that prostatectomy without ADT may weaken the antineoplastic effect of metformin. According to our subgroup analyses, ADT with metformin improves PCa-specific survival and overall survival. However, ADT increases the incidence of metabolic syndrome, such as obesity, hyperinsulinaemia, insulin resistance and type-2 diabetes mellitus^27^. Given that metabolic syndrome is an important factor for biochemical failure after prostatectomy and radiotherapy, metformin exhibited therapeutic benefits for weight gain induced by medications and metabolic disturbances related to insulin resistance^28–30^. In benign prostate hyperplasia (BPH) xenograft models, metformin inhibits testosterone and attenuates prostate weight and pathological alterations^31^. These findings suggest that metformin not only reduced the side effects of ADT but also acted as chemotherapy for ADT through testosterone inhibition. Docetaxel is a first-line chemotherapy for treating castration-resistant prostate cancer (CRPC). Hyperglycaemia, which is a side effect of docetaxel, reduces the efficacy of docetaxel at inducing PCa cell apoptosis^32^. Biernacka *et al*. demonstrated that co-treatment with docetaxel and metformin led to additive effects to induce PCa cell apoptosis and alleviated the resistance induced by hyperglycaemia^33^. However, only two clinical studies examined the relationship between docetaxel and metformin^16,34^, and none of these studies revealed that metformin exhibited an additive effect with docetaxel. Further clinical studies are needed to discover whether metformin therapy could improve the prognosis of both PCa and CRPC.

Coinciding with the prognosis of PCa, the association between metformin therapy and PCa incidence is controversial. Bansal *et al*.^10^ demonstrated that diabetes reduced the diagnosis of PCa by 14% compared with those without diabetes. Some studies noted that compared with non-diabetic patients, diabetic patients exhibited reduced levels of testosterone, which decreased the incidence of low-grade PCa^11,35–37^. However, Azoulay *et al*.^38^ summarized data from the UK General Practice Research database and found that metformin intake increases the incidence of PCa. Various studies reported conflicting results with the association of metformin usage and PCa diagnosis^39–43^. To clarify this association, we included 12 studies and found no association between metformin usage and the incidence of PCa. A previous meta-analysis provided similar results between PCa risk and metformin exposure^44^. In contrast, two studies^45,46^ reported slight reductions (12% and 9%) in PCa risk and metformin use with substantial heterogeneity. (*I*^2^ = 74.7% and 51%). In their meta-analysis, all the included studies were published earlier than 2014. However, our study identified 12 studies and included 1,431,979 male subjects, a larger population group than previous studies^45,46^. Moreover, more than 92% studies were published in the past five years. Therefore, our results gained stronger statistical power.

PCa occurrence and outcomes vary considerable between racial and ethnic groups. Siegel *et al*.^1^ reported that PCa incidence and mortality are generally highest among American Africans, whereas Asians exhibited the lowest PCa rates. However, compared with non-Asian patients with type 2 diabetes, Asian patients with type 2 diabetes exhibit a significantly increased risk of PCa^10,47^. One large population-based study reported that Hispanics undergoing metformin therapy exhibited an evident reduction in PCa incidence, whereas metformin usage is not associated with PCa incidence among African Americans and non-Hispanic whites^48^. Previous meta-analyses on this topic revealed no association between metformin and incidence of PCa in either Western-based or Asian-based populations^49^. However, Western and Asian populations were only classified based on geography, and this studies were limited by significant heterogeneity (*I*^2^ = 88%). Unlike the previous study, we classified all participants as African American, Latino/Hispanic, Non-Hispanic white and Asian. We found that metformin use is associated with a 14% reduction in the risk of PCa among non-Hispanic whites with the presence of heterogeneity (*I*^2^ = 49%). However, metformin therapy did not decrease the risk of PCa among American Africans (*I*^2^ = 26%). A high degree of heterogeneity was noted among Hispanics/Latinos and Asian (94% and 100%, respectively). This high heterogeneity is consistent with a previous study^50^. We found that this evidence heterogeneity is heavily influenced by the studies of Raval and Tseng, which were large studies with an extreme risk estimate. Given that there were only two studies in each subgroup, these studies also had a high level of precision and a high Newcastle-Ottawa score, we included this study. In other subgroup analyses, duration of metformin therapy, cumulative metformin dose, and study region exhibited no association with the incidence of PCa. Moreover, we found that metformin usage was not associated with the Gleason scores of PCa.

There was evidence of considerable heterogeneity in OS (*I*^2^ = 89%, *P* < 0.00001), CSS (***I***^2^ = 67%, ***P*** = 0.006), RFS (***I***^2^ = 63%, ***P*** = 0.009) and incidence of PCa (*I*^2^ = 98%, *P* < 0.00001). A sensitivity analysis found that Zaosky 2017 and Tseng 2014 were the sources of heterogeneity in the meta-analysis for RFS and PCa risk. The study by Zaorsky 2017 failed to report the exact start and stop times of metformin. Information on the timing and amount of metformin use was unclear, which might cause a time bias and lead to heterogeneity. In Tseng 2014, no information was available on lifestyle variables, such as smoking status, alcohol consumption, or diet, that potentially influenced the risk of PCa. Although 4 studies^39,41,43,49^ also fail to record the lifestyle in the group of incidence. Differing from these studies which lack information on lifestyle factors, Tseng only focuses on the local Asian administrative databases. We suppose that the lack of information on lifestyle factors in Asian may be the potential reason for heterogeneity in Tseng *et al*. Moreover, there are only two studies which focus on the Asian in the group of incidence. We use subgroup analysis and found that these two Asian studies also have a high degree of heterogeneity (***I***^2^ = 100%). The participants in Tseng *et al*. were from the National Health Insurance reimbursement database, which is a local Asian database in Taiwan.While in Chen *et al*.^49^, the participants were Asian Canadian and come from British Columbia Cancer Agency in Canada. This difference of such criteria included may also lead to the heterogeneity in Tseng 2014. The Newcastle-Ottawa score revealed that these two studies were not more biased compared with other studies and had large sample sizes. Therefore, it is inappropriate to exclude these two studies from our meta-analysis.

There were various strengths of our meta-analysis. First, we comprehensively searched relevant studies using Embase, PubMed and Cochrane without publication date or publication type limits by extracting the maximal number of dates in suitable studies. Second, a total of 30 cohort studies including 1,660,795 individuals were included in our studies, which allowed us to quantitatively assess the relationship between metformin intake and PCa. Third, various subgroup analyses, such as PCa treatment, race, duration of metformin therapy, cumulative metformin dose and PCa Gleason score, could provide precise evidence for metformin use in clinical practice. Fourth, we only included the patients with metformin monotherapy and reduced the anticancer bias of other medications.

There were some limitations of our meta-analysis. First, two studies^51,52^ did not report the number of metformin users and non-metformin users, and 1 study^53^ did not separate type 1 and type 2 diabetes, which may affect the accuracy of the final result. Second, the accuracy of the summary estimates is influenced by different survival analysis methods. Although a multivariate Cox proportional hazards model was used in most of the studies, 2 studies^54,55^ did not report their statistical models. Third, because our meta-analysis exclusively focused on studies written in English, a language bias might exist. Fourth, most of our included studies were retrospective studies, which affected the quality of evidence for our meta-analysis.

In conclusion, our meta-analysis suggested that metformin therapy exhibits advantages in improving the prognosis of PCa, but no association was noted between metformin usage and PCa incidence. Moreover, PCa patients with metformin therapy accepting radical radiotherapy exhibited more dramatic effects on OS, CSS and RFS. These studies demonstrated a useful direction for the clinical treatment of PCa. Further randomized controlled trials are needed to confirm the association of PCa and metformin usage.

## Materials and Methods

### Study selection

Two authors (He & Hu) performed an electronic search of the PubMed, Embase, and Cochrane databases for relevant English studies (the last search update was May 20, 2018). The search strategies included ‘metformin’, ‘biguanide’, ‘Dimethylbiguanidine’, ‘Prostate cancer’ and ‘Prostate Neoplasms’. All the included studies met the following criteria: 1) Study designs must be prospective or retrospective cohort study. Studies must compare metformin users and non-metformin users. 2) Studies must analyse the PCa incidence, overall survival (OS), PCa-specific mortality (CSS) or recurrence-free survival (RFS). We excluded the following types of studies: reviews, case-control studies, studies of interventions other than metformin, articles assessing outcomes following metformin use in animal models, metformin use in other populations, studies including metastatic PCa patients at diagnosis and *in vitro* studies. Language selection focused on articles written in English. Hazard Ratio (HR) was used as the measure across studies. Given that the PCa incidence was relatively low, odds ratio (OR) were used as an estimate of HR. The prognostic outcomes estimate HRs/RRs with 95% CIs. RFS was defined as the time from the date of PCa patients accepting prostatectomy, radiation therapy or androgen deprivation therapy to the date of biochemical recurrence. After removing duplicate publications, two authors (He & Hu) independently assessed the primary literature by assessing titles and abstracts and then identified the final relevant studies based on eligibility.

### Data extraction and Quality assessment

Two authors (He & Ye) extracted data and information from final studies, such as the first author, year of publication, study region, sample size, study design, follow-up period, type of treatment, and survival endpoints. Two authors (He & Ye) assessed the final studies, scored them using the NOS^56^ and reached a consensus value for each study independently.

### Statistical analysis

Review Manager 5.3 (RevMan 5.3) was employed to conduct all statistical analyses. PCa incidence and survival estimates were abstracted from the final studies and pooled using a random-effects model. Standard Cochran’s Q test and ***I***^2^ statistics were used to identify heterogeneity between the included studies. A value of ***I***^2^ statistics >50% and *p*-value <0.1 indicated significant heterogeneity. When heterogeneity was significant, we explored the potential influential variables between included studies and pooled the results into subgroup analyses. Publication bias was detected with the Begg and Egger’s regression intercept test by using STATA 13. (Stata Corp LP, college Station, TX).

### Ethics approval

Ethical approval was not sought as the study was based entirely on previously published data.

## Data Availability

The study was based entirely on previously published data.
